# Families with Internationally Adopted Children in Finland: A Study of Emotional Availability in the Early Interaction

**DOI:** 10.1007/s10578-024-01769-0

**Published:** 2024-10-15

**Authors:** Katarina L. Kuusela, Hanna Raaska, Marko J. Elovainio, Anna-Riitta Heikkilä, Christian A. Hakulinen, Marjo S. Flykt, Helena Lapinleimu

**Affiliations:** 1 Department of Child Psychiatry, University of Helsinki, PO BOX 63, 00014 Helsinki, Finland; 2 Department of Child Psychiatry, Helsinki University Hospital, PO BOX 22, 00014 Helsinki, Finland; 3 Department of Psychology and Logopedics, University of Helsinki, PO BOX 63, 00014 Helsinki, Finland; 4 Department of Pediatrics, University of Helsinki, PO BOX 63, 00014 Helsinki, Finland; 5 Department of Pediatrics, Helsinki University Hospital, PO BOX 22, 00014 Helsinki, Finland; 6 Finnish Institute for Health and Welfare, PO BOX 30, 00271 Helsinki, Finland; 7 Department of Paediatrics and Adolescent Medicine, University of Turku, 20014 Turun Yliopisto, Finland; 8 Department of Paediatrics and Adolescent Medicine, Turku University Hospital, 20014 Turun Yliopisto, Finland; 9 The Social Insurance Institution of Finland, PL 10, 00056 Helsinki, Kela Finland; 10 Faculty of Social Sciences/Psychology, Tampere University, 33014 Tampere, Finland

**Keywords:** Internationally adopted children, FinAdo, Early interaction, Emotional Availability, Parenting

## Abstract

A well-functioning parent–child relationship is crucial for the child’s psychological development. We examined the Emotional Availability (EA) in the early interaction of internationally adopted children with their mothers. We also studied whether the quality of the interaction was associated with the sex of the adopted children, the age at the time of adoption, the time they had spent in the family and parental depressive symptoms. The study sample was part of the Finnish Adoption (FinAdo) study and included 79 children (mean age at adoption = 2.58 years, SD = 1.51 months, 37% girls) and their adoptive mothers in Finland. The mother–child interactions were examined with Emotional Availability Scales (EAS) during the first months after adoption (mean = 6.3 months). Our results showed that the overall EA scores were relatively high (mean 4.78–6.18), although the mean levels of parent sensitivity, parent non-intrusiveness, child responsiveness and child involvement were under the high zone of the EA (< 5.5). Children adopted at a younger age and boys received lower scores in the interaction analysis. Our results suggest that families with internationally adopted children seem to be able to create a well-functioning early relationship between the mother and the child.

## Introduction

Internationally adopted children represent a population at developmental risk, considering their potential history of early adversities and deprivation. The children may have pre- and post-natal risk factors as well as maltreatment and neglect in their background, creating a mixture of negative experiences affecting their development [1, 2]. The absence of a stable caregiver is a common feature in the background of these children as they typically have experienced institutional rearing, placing the children at risk for various psychopathology in both their socioemotional and physiological development [3–5]. The lack of a long-term relationship with a sensitive caregiver may predict disturbances in attachment patterns [4, 5], increasing risk for later emotional problems [6, 7]. However, the transition of a child from institutional care into an adoptive family can lead to a significant recovery [5, 8] and studies suggest that children begin to form an attachment relationship with the adoptive parents during the first six months after arriving into the family [9–12]. According to the transactional model of development, the child and the parent mutually influence one another during the development of their relationship [13]. Internationally adopted children have pre-adoptive experiences that influence the forming of a new relationship with their adoptive parents. Examining this parent–child interaction in more detail and the potential effect of the age when the process starts and the sex of the child in this process in adoptive families can give us more understanding and clinical perspective in this relationship in the first months after adoption.

There are studies about the impact of sensitive parenting on attachment after adoption [9, 14–16] as well as about the impact of different aspects of parenting, including sensitivity, structuring, consistency, efficacy and lack of intrusiveness, on the emotional and behavioral adjustment of the adoptive child [15, 17, 18]. Studies have demonstrated the effect of the internal working models of the adoptive mother on early attachment patterns of the adoptive child [11] as well as the mediating effect of attachment security on later psychopathology of children having a background in institutions [19]. Many studies have concentrated only on parental dimensions of the relationship of the adoptive child with the parent, although the effects of child individual characteristics on the interaction may be specifically important in adopted children, considering the large amount of institutional background and potential history of neglect among them. It has been also argued that internationally adopted children may represent a population with atypical attachment behavior because of their exceptional background in institutional rearing conditions, suggesting methodological difficulties in assessing attachment in this population [4, 10]. Studies on early attachment development of adopted children have found mixed results in terms of security of attachment [11, 12, 20]. Thus, more understanding in specific behavioral patterns of the child as well as in attachment development in this early relationship is needed.

The Emotional Availability Scales (EAS) were developed from the theory of Emotional Availability (EA) that has its background in attachment theory as well as several other theoretical influences like the transactional model, the systems theory, the emotion theory and the psychodynamic theory [21]. The EA includes four dimensions for the parent (sensitivity, structuring, non-intrusiveness, non-hostility) and two dimensions for the child (responsiveness, involvement of the parent) to describe the behavior and the EA of the parent–child dyad in the interaction [21, 22]. Biringen and colleagues describe the EA: “All these dimensions are dyadic or relationship constructs, such that the adult´s and child´s behaviors are viewed in the context of a dynamic, mutually reinforcing feedback loop, rather than only as an individual´s behavior” [23, p. 192]. Furthermore, the EA is described to be an overlapping, but not identical construct with attachment, measuring emotional attachment of the relationship [23, 24]. Whereas the focus of the traditional attachment measures is in the ability of the child to rely on the parent during stress, emotional attachment includes the affective expression and emotional connection of the relationship that occur also outside of stressful situations [23, 24]. There are studies showing association between the EA and attachment [25, 26], and the adult sensitivity scale and the child responsiveness scale have been validated against insecure/secure attachment [24]. Studies have shown association between the EA and several aspects of socioemotional development in non-adopted children [27–29] as well as between child-related risk factors (developmental delay, very low birth weight/pre-term children) and the child EA [30, 31]. Thus, examining early interaction of internationally adopted children using all the dimensions of the EA may give us more understanding in specific patterns of behavior that reflect socioemotional development of these children.

There are studies using the EA in assessing the early parent–child interaction of adoptive families [32, 33]. In these studies higher EA was associated with better social functioning and less indiscriminate friendliness and moreover, former foster children presented a larger increase in responsiveness than post-institutionalized children in the first 6 months after adoption. In the study by van den Dries and colleagues, no association was found between attachment security and the EA dimensions child responsiveness and adult sensitivity. Two studies have examined the effect of a parenting programme on socioemotional adjustment of the child using the EA, and both studies found a significant increase in the EA after intervention attendance [34, 35]. One study using the EA in the interaction analysis found that the EA dimensions were associated with child and parent attachment security [36]. Only two of these studies have used all the EA dimensions in the analysis and these did not examine interaction in the early stage after adoption (mean time spent in families 19–41 months) [34, 36]. The studies examining interaction in the first months after adoption either used only some of the dimensions [33], or summarized different dimensions of EA together [32, 35], and none of the studies have used the child involvement scale. Hence, to our knowledge this is the first study that has analyzed the quality of early interaction of internationally adopted children with their adoptive parents using all the EA dimensions separately.

In addition to the impact and quality of the early post-adoption interaction, it is interesting how child-and parent-related factors influence this interaction. Studies suggest that age at adoption, duration of institutionalization and quality of care in institution are risk factors for socioemotional development of internationally adopted children [8, 20, 37]. It has been suggested that children who have experienced more pre-adoptive adversity receive lower-quality early post-adoptive parenting and parent–child interaction [18, 32]. A meta-analysis on the attachment relationships of adopted children showed that adoption after their first birthday predicted less attachment security in adopted children [8], whereas in the Bucharest Early Intervention Project, Smyke and colleagues found that children adopted before 24 months of age were likelier to have secure attachments compared with children who were older at adoption [37]. Furthermore, studies on non-adopted children have shown that maternal depression and lower socioeconomic status of the family predict lower-quality parenting and interaction [38, 39]. In terms of sex of the child, early-onset disorders, such as conduct disorders and neurodevelopmental disorders, show a male preponderance [40]. From a transactional model perspective, it may be predicted that psychiatric symptoms of the child may have an impact on the interaction with the parent and vice versa and it has been proposed that one mechanism to sex differences in children’s behaviour could be that parents may treat boys and girls differently [40]. A study examining EA of non-adopted children with their parents found that the mother-daughter dyads displayed higher EA than the mother-son dyads [41]. The study also revealed that the father-daughter dyads showed lower EA than the mother–child dyads and the father-son dyads scored the lowest. There are some studies on internationally adopted children showing that boys have more psychiatric symptoms than girls [19, 42], and to our knowledge one study has found a difference in parenting (parent summary EA score) between boys and girls [32].

The aim of the current study was to examine the early relationship between mostly post-institutionalized internationally adopted children and their adoptive mothers at the time of transition into an adoptive family. First, we hypothesized that the child dimensions would be lower than the high zone of the EA, as suggested by previous studies [33, 35]. Second, we hypothesized that most of the adult dimensions would be in the high zone of the EA, as suggested by previous studies [32, 33, 35]. We also examined the impact of risk factors on the early relationship of adoptees, as there are limited studies on this subject. We examined the impact of (a) age of the child at the time of arrival in adoptive family, (b) time that the child had spent in the family, (c) child sex assigned at birth and (d) depressive symptoms of the mother. Our third hypothesis was that the quality of the interaction would be decreased by (1) older age at the time of adoption, (2) less time spent in the family, (3) the child being a boy and (4) more depressive symptoms of the mother.

## Methods

### Participants and Procedure

The study is part of the ongoing Finnish Adoption (FinAdo) study. The target population of this study consisted of internationally adopted children under the age of seven who came to Finland between October 2012 and December 2016. The children were adopted through three authorized adoption organizations in Finland. The mean time from arriving in the family to the assessment was 6.3 months. Background information, including child sex, age at adoption, continent of birth and number and type of placements before adoption, as well as parent age, parent gender, marital status of the parent and socioeconomic status of the family, was gathered with self-administered questionnaires in the first months after adoption in connection with the recording of interaction. The characteristics of the sample are presented in Table 1. In the recruitment, we asked the primary caregiver of the child to attend the interaction analysis. The study initially included 86 internationally adopted children and their parents, with 7 father-child dyads and 79 mother–child dyads attending. Due to the small number of father-child dyads, it was not possible to analyze gender differences in the interaction. As the primary caregiver of the child in Finland is still nearly always the mother [43], we only included the mother–child dyads in the study. In previous studies that have examined EA in adopted and non-adopted children, sample sizes have been similar [29, 31–33, 35]. In this study, most of the children were boys and the mean age at the time of adoption was 2.5 years. As for continent of birth, Asia and Africa were overrepresented, as opposed to Eastern Europe and South America. The only additional information available about the children before adoption was the type and number of placements.

**Table 1:** Characteristics of the study sample

Children	
Girls	29 (37%)
Boys	50 (63%)
Continent of birth	
Asia	38 (48%)
Africa	30 (38%)
South America	5 (6%)
Eastern Europe	6 (8%)
Age at arrival in Finland in years, mean (SD)	2.52 (1.38)
Time spent in the family in months, mean (SD)	6.3 (3.7)
Type of placement	
Orphanage	59 (75%)
Foster family/relatives	3 (4%)
2–3 placements	6 (7%)
Several placements	7 (9%)
Unknown*	4 (5%)
EAS dimensions of the children	
Child responsiveness, mean (SD)	4.91 (1.37)
Child involvement, mean (SD)	4.78 (1.26)
Categorical EAS of the children	
*EAS 5.5 or over*	
Child responsiveness	31 (39%)
Child involvement	33 (42%)
*EAS under 5.5*	
Child responsiveness	48 (61%)
Child involvement	46 (58%)
Parents	
Socioeconomic status of the family	
Low	4 (5%)
High	57 (72%)
Unknown*	18 (23%)
Psychological distress (GHQ)	
Total, mean (SD)	2.11 (2.75)
Anx / Dep (1), mean (SD)	1.08 (1.25)
Soc. Dys. (2), mean (SD)	0.84 (1.46)
Loss of Conf. (3), mean (SD)	0.19 (0.54)
EAS dimensions of the parent	
Parent sensitivity, mean (SD)	5.38 (1.05)
Parent structuring, mean (SD)	5.53 (1.11)
Parent non-intrusiveness, mean (SD)	5.48 (1.32)
Parent non-hostility, mean (SD)	6.18 (1.15)
Categorical EAS of the parent	
*EAS 5.5 or over*	
Parent sensitivity	43 (54%)
Parent structuring	44 (56%)
Parent non-intrusiveness	53 (67%)
Parent non-hostility	65 (82%)
*EAS under 5.5*	
Parent sensitivity	36 (46%)
Parent structuring	35 (44%)
Parent non-intrusiveness	26 (33%)
Parent non-hostility	14 (18%)

Figures are frequencies and percentages unless specified

*EAS* Emotional Availability Scales; *GHQ* General Health Questionnaire; *Anx* Anxciety; *Dep* Depression; *Soc. Dys.* Social Dysfunction; *Loss of Conf* Loss of Cofidence; *SD* Standard deviation

^*^Parent did not report

The study was approved by the ethics committee of the Hospital District of Southwest Finland. Written and informed consent was obtained from the parents.

### Measures

*Emotional Availability Scales.* The EAS were used to examine the mother–child interaction and assessed during a 20 min play interaction between the mother and the child that was videotaped. For the first five minutes, the mother–child dyad was given a structured task, which was a puzzle or a book to read. Afterwards, they were instructed to play as they normally would for 15 min, with toys provided. All recordings took place in the same laboratory environment.

The EAS established by Biringen aim to assess the emotional quality of caregiver-child interactions [21, 22]. There are six dimensions (scales) assessed separately using a 7-point rating scale: parental sensitivity, parental structuring, parental non-intrusiveness, parental non-hostility, child responsiveness to the parent and child involvement of the parent. The EAS are described as a relationship construct so that the emotional availability of both partners is evaluated in the context of the observed relationship [21, 22]. This means that the responses and qualities of the other part are considered when the other part is assessed, so that the six dimensions are seen as descriptions of the interaction rather than describing individual behavior. Parental sensitivity is described as optimal when creating a positive and genuine affective climate. Sensitivity refers to good perception of the expressions of the child and appropriate responsiveness, attunement to timing and rhythm, flexibility, variation, creativity and acceptance of the child. Parental structuring is described as a framework that the parent provides for the child with appropriate limits and guidance while maintaining a sense of autonomy. As for non-intrusiveness and non-hostility, the high end of the dimensions is always optimal, meaning the absence of a negative quality. Non-intrusiveness is described as lack of over-direction, over-stimulation, interference or over-protection, whereas in optimal non-hostility there are no signs of overt or covert hostility. Responsiveness of the child to the adult is optimally reflected as good affect and responsiveness whenever the caregiver invites the child into interaction, whereas the child involving the parent refers to the ability of the child to involve the caregiver in the interaction.

For all the dimensions, the scorings can be categorized in the high zone (scores from 5.5 to 7), the moderate zone (scores from 4 to 5), the low zone (scores from 2.5 to 3.5) and the problematic zone (scores from 1 to 2) [22]. Within the parent sensitivity and child responsiveness scales, the scorings can also be categorized in the emotionally available zone (scores from 5.5 to 7), the complicated zone (scores from 4 to 5), the detached zone (scores from 2 to 3.5) and the problematic/disturbed zone (scores from 1 to 2) and these zones can be viewed as a measure of emotional attachment [23, 24]. For these emotional attachment zones, all the zones under the emotionally available zone represent insecure emotional attachment [23, 24]. In this study, we used the scoring of the EAS as a dimensional variable and as a categorical variable. For the categorical variable, we classified the scoring into two categories, under 5.5 points and 5.5 points or over, which has been used in previous studies [28, 38, 44], although use as a dimensional variable is most common. Both dimensional and categorical scorings were used in the regression analyses.

Many studies have suggested acceptable validity and reliability for the EAS [24–26, 45–49]. One study examining the short-term reliability and continuity of the EAS in mother–child dyads found reliabilities that ranged between 0.79 and 0.92 [47]. Another study reported longer-term stability in assessing mother–child dyads at child ages of 18 months, 24 months and 36 months [46]. The EAS were stable between the first two assessments at home but no longer in the last lab assessment.

The analysis of the interaction recordings was assessed by one trained coder, who was approved as reliable to code by Biringen (Colorado State University, US). Reliability was tested with another trained and reliable coder, assessing approximately 20% of the recordings. The intra-class intercoder reliabilities for randomly chosen observations were 0.81 (parent sensitivity), 0.84 (parent structuring), 0.79 (parent non-intrusiveness), 0.64 (parent non-hostility), 0.79 (child responsiveness) and 0.87 (child involvement). The low intra-class reliability for parent non-hostility was regarded as being caused by small variations in the scorings. Most of the scorings of this dimension were classified in the high zone (5.5–7); therefore, we also used this dimension in the analysis.

*Child-related and family-related risk factors.* Data were gathered via a questionnaire developed for the FinAdo study to gain knowledge about the characteristics of the child and the adoptive family. The child-related variables included the child’s sex, age at the time of adoption, continent of birth and number and type of placements before adoption. The family-related variables included age, gender and marital status of the parent as well as socioeconomic status of the family.

*Parental depressive symptoms.* The General Health Questionnaire (GHQ) is a widely used questionnaire for assessing psychiatric morbidity [50]. There are several shorter versions of the original 60-item version (30, 28, 20 and 12 items). In our study, we used the short version with 12 items, which has been found to detect anxiety and depression, social dysfunction and loss of confidence in confirmatory factor analyses [51] and it has shown good validity for measuring depression [52]. The questions were answered on a 4-point scale: 1 = more than usual, 2 = as much as usual, 3 = less than usual and 4 = much less than usual. We used the total GHQ score as well as the three subscales for anxiety and depression, social dysfunction and loss of confidence.

### Statistical Analyses

The child’s age was analyzed as a continuous and a categorical variable (under two years and over two years), and the time spent in the family was classified into two categories (under six months and over six months). We had to combine Eastern Europe and South America as one group due to the low amount of children adopted from these continents, so that the child’s continent of birth was classified into three categories (Africa, Asia, Other = Eastern Europe and South America). For the GHQ scores, we used the four different categories (total score and the three subscales) together and each of the three subscales separately. Girls were used as reference for the sex of the child and Asia was used as reference for continent of birth.

We examined differences in the outcomes between boys and girls and between two different age groups (under two years and over two years) using Student’s t-test. The association between EAS and sex of the child, age of the child (as continuous variable) and continent of birth was analyzed using multivariable linear regression analyses. The association between GHQ and EAS were examined using linear and logistical regression analyses. The association between EAS and the time the child spent in the family was tested using a linear regression model. The results in linear regression models were adjusted for age and sex of the child as well as continent of birth. All analyses were conducted using Stata 17.0.

## Results

Table 1 shows the mean values for the EAS and GHQ scores. Mean values for parent sensitivity, parent non-intrusiveness, child responsiveness and child involvement were under the high zone (< 5.5 points). The mother–child dyads were also divided between the two categories of the six dimensions of the EAS scorings (Table 1). Most of the children’s scores were under the high zone (< 5.5 points) whereas the majority of the parental scales were in the high zone (≥ 5.5 points).

Table 2 shows the differences in outcomes between older and younger participants, as well as between girls and boys. Parent sensitivity, parent structuring, parent non-intrusiveness and child involvement were higher in the older age group. Parent non-hostility was higher in girls.

**Table 2:** Differences in the EAS outcomes between the two age groups and between girls and boys

	The two age groups					Sex				
EAS dimension	< 2 years		> 2 years			Girls		Boys		
	M	SD	M	SD	p	M	SD	M	SD	p
Parent sensitivity	5.02	1.21	5.61	0.98	0.02	5.59	1.09	5.24	1.13	0.19
Parent structuring	5.17	1.20	5.77	1.05	0.02	5.64	1.22	5.45	1.11	0.49
Parent non-intrusiveness	5.00	1.52	5.89	1.08	0.00	5.86	1.29	5.35	1.36	0.10
Parent non-hostility	5.88	1.29	6.34	1.03	0.08	6.55	0.83	5.90	1.26	0.02
Child responsiveness	4.56	1.52	5.10	1.25	0.09	5.21	1.37	4.68	1.38	0.11
Child involvement	4.42	1.45	5.00	1.07	0.05	5.00	1.19	4.62	1.31	0.21

*EAS* Emotional Availability Scales; *M* mean; *SD* Standard deviation

In the combined regression model, there was an association between the age of the child and EAS, as well as the sex of the child and EAS (Table 3). The older age of the child was associated with higher parental scores in non-intrusiveness, whereas the scores in parent non-hostility were higher in girls. Continent of origin was not associated with EAS, and the differences that were found between the two age groups in parent sensitivity, parent structuring and child involvement were not statistically significant in the combined regression model. In the linear and logistical regression models, we found no significant associations between the EAS and GHQ, nor between EAS and time spent in the family (results not shown; all p-values > 0.05).

**Table 3:** The association between age, sex and continent of birth with EAS dimensions (univariable associations)

	Age			Sex			Continent of birth		
	b	SE	p	b	SE	p	b	SE	p
Parent sensitivity	0.18	0.09	0.06	− 0.36	0.26	0.16	0.20	0.19	0.29
Parent structuring	0.19	0.10	0.06	− 0.19	0.26	0.47	0.18	0.20	0.35
Parent non-intrusiveness	0.26	0.11	0.02	− 0.53	0.30	0.09	0.01	0.22	0.95
Parent non-hostility	0.11	0.09	0.25	− 0.63	0.26	0.02	− 0.09	0.19	0.63
Child responsiveness	0.13	0.12	0.28	− 0.53	0.32	0.10	0.27	0.24	0.25
Child involvement	0.15	0.11	0.16	− 0.39	0.29	0.19	0.22	0.22	0.31

*EAS* Emotional Availability Scales; *b* Beta coefficient; *SE* Standard error; References: *Sex* girls; *Continent of birth* Asia

## Discussion

We examined the early interaction between mostly post-institutionalized internationally adopted children and their mothers at this time of transition. We also studied risk factors influencing this early interaction. Our results revealed that most of the child scores in the EA dimensions were lower than the high zone of the EA dimensions 6.3 months (mean) after adoption, and the mean values of the child scores were under this zone. Most of the parental scores of the EA dimensions were in the high zone of the EA. Mean values of parent sensitivity and parent non-intrusiveness were under but very near to the high zone, and moreover, age and sex of the child had a significant effect on parent dimensions. To our knowledge, no previous study has examined interaction of internationally adopted children with their mothers in the early stage after adoption using all the EA dimensions separately, including two child dimensions.

As hypothesized, the mean scores for child responsiveness and child involvement were under the high zone of the EA in this study. The few previous studies on internationally adopted children and their early responsiveness found similar scores to our study [15, 33]. A study by Barone et al. that combined child responsiveness and child involvement in one score found similar scores [35]. In their short-term longitudinal study, van den Dries et al. examined the effect of pre-adoptive care on child responsiveness in internationally adopted girls from China (mean age at adoption = 13 months, analysis of interaction = 2 months and 6 months after adoption) [33]. Post-institutionalized and former foster children did not differ in responsiveness, but former foster children presented a larger increase in responsiveness than did post-institutionalized children. In our study, the mean time the children had spent in their adoptive families was 6.3 months. Having a background mostly in institutions, the result of our study on child responsiveness is in line with the van den Dries study, as the level of the child responsiveness score in our study was like the post-institutionalized group at six months after adoption. This may reflect the effect of the more depriving backgrounds in institutions to the socioemotional capacities of these post-institutionalized children. Our study adds to these studies because we analyzed both child responsiveness and child involvement as two separate components. According to our results, the way most children involve their mothers at this time point is under the high zone of the involvement dimension. In the EA, child behavior is described at various levels, avoidant or involving the parent in negative ways when the involvement dimension is scored under 5.5 points [22]. This behavior may be understandable considering that the child has spent a relatively short time in the new family and also considering post-institutionalized internationally adopted children being a population with many potential risk factors that are often mostly unknown. One study found that children with very low birth weight and pre-term children scored lower in the both child dimensions of the EA than full-term children with normal birth weight [31]. A study in non-adopted children (average age 31.97 months) with major social impairment found that higher developmental level was associated with higher child responsiveness and child involvement [30] and in another study in non-adopted children with autism spectrum disorder it was found that the child EA dimensions were associated with their cognitive functioning and their severity of symptoms 53. Both child responsiveness and child involvement have been shown to be predictors for child empathy toward the mother and toward an adult outside the relationship in non-adopted children [54].

The majority (61%) of the children in this study showed insecure emotional attachment as scores under 5.5 in the child responsiveness scale are classified as being in insecure emotional attachment zones in the EA [24]. In previous studies on attachment of adopted children, post-institutionalized children have shown to be as securely attached to their adoptive parents 6–9 months after adoption as non-adopted children [12, 20] whereas other studies have shown significantly or slightly less secure attachment and more disorganized attachment compared to normative population 6 months after adoption [11, 33]. Our results may reflect the ongoing development of children’s internal working models in the first months after adoption. It has been argued that the behavior of post-institutionalized children in the first months after adoption that has been classified in insecure attachment categories may in fact represent reorganization of their attachment patterns and organization of their socioemotional capacities for the first time [10, 20, 33]. In this light, this behavior may rather be seen as a phase in development than an organized attachment strategy or a breakdown in strategy [20, 33]. Our study may give us a new perspective in understanding the development of early attachment patterns as the child responsiveness scale is described to show the emotional attachment style of the child [23, 24]. According to our results, most of the adopted children still haven’t been able to form a secure emotional attachment or may be in the process of reorganizing and developing new emotional attachment strategies. In future, longitudinal studies are needed for further understanding in this process.

Our second hypothesis was supported as most of the scores for the parent dimensions were in the high zone of the EA and also the mean values of these dimensions were in or very near this zone. Most of the adoptive mothers were classified in the secure emotional attachment zone and were emotionally available to their adoptive child as the sensitivity score was 5.5 or over for most of the mothers. Our results are in line with previous studies showing that early parenting in families with internationally adopted children with a background in institutions has received relatively high scores [17, 18, 32]. In a study by Lawler and coworkers, lower-quality pre-adoptive care and more pre-adoptive deprivation were associated with lower-quality parenting in the group of post-institutionalized children [18]. Lawler et al. examined parent sensitivity and structuring in the interaction, whereas our study included parent non-intrusiveness and parent non-hostility in the analysis. Studies have shown that early good-quality parenting may have a protective effect on several aspects of later socioemotional development, especially for internationally adopted children with a background in deprivation [18, 32, 35, 55, 56, 33]. Even small variations in parenting may be meaningful during this period of rapid development observed in the months following removal from institutional care. The impact of sensitive parenting is widely known to be crucial for the development of both non-adopted and adopted children [9, 14, 33, 34, 55]. In addition to sensitivity, parental non-intrusiveness has been connected to better socioemotional development of the child [32, 57–59]. One basic element in non-intrusiveness within the EA is parental ability to support child autonomy [22], which has been connected to security of attachment, better executive function and less internalizing symptoms in non-adopted populations [57–59], as well as better emotional knowledge in internationally adopted children [32]. Thus, considering the importance of parental non-intrusiveness may provide a better understanding of the early interaction in the first months after adoption. Our study suggests that the adoptive mothers were able to structure their adoptive children well in a non-hostile manner. Structuring has been connected to better socioemotional development in internationally adopted children [17, 18] and furthermore, several aspects of parental hostility have been associated with predicting child psychopathology in non-adopted children [60, 61]. At this time point, the mothers may still be in the adaptation process into the mother–child relationship with their newly adopted child and more study is needed on the development of the interaction and parenting.

Age at adoption was a predictor of scores for parent non-intrusiveness. Contrary to our hypothesis, the scores were higher in the older children. In studies, older age at adoption has been associated with less attachment security and more persistent symptoms of disinhibited social engagement disorder [5, 8]. Moreover, older age at adoption has been shown to predict more emotion regulation problems three months after adoption and more internalizing and externalizing psychiatric symptoms in the years following adoption [18, 42, 62]. In terms of parenting, older age at adoption has been associated with lower-quality structuring 11 months after adoption [18] and less supportive parenting some years after adoption [63]. In our study, lower parental non-intrusiveness in younger children may reflect the challenging situation of parenting a child with a background in deprivation. According to our results, in the first months after adoption, mothers may be in a more difficult position with children adopted at a younger age. One explanation could be that mothers may have more difficulties in interpreting a younger newly adopted child which may lead to more intrusive behaviour from the mother. According to the studies mentioned above [18, 63], interaction in families with children adopted at younger age is later in development of higher quality than in families with children adopted at older age. Further study on the development of this interaction is needed, as it could be proposed that younger adoptive children may rapidly develop soon after adoption in improving their ways of interacting with their parents.

Parental non-hostility was lower in boys than in girls, as expected in our hypothesis. Studies on child sex and parenting in adopted and non-adopted children have found mixed results [32, 64–66]. In the studies on internationally adopted children and children adopted at birth, it was found that boys received lower quality parenting (parent summary EA score) and more negative parenting behaviors than girls [32, 67]. In studies on non-adopted children, a meta-analysis of the effect of child sex on parents’ use of autonomy-supportive and controlling strategies found minimal differences between boys and girls [64], whereas some other studies have found that boys tend to receive more physical punishment than girls [65, 66]. Our study contributes to these studies with the setting of international adoption and with no genetic background between the parent and the child. In studies on internationally adopted children, it has been found that boys had more symptoms of indiscriminately social/disinhibited reactive attachment disorder, more symptoms of externalizing disorders, more symptoms of internalizing disorders and more functional impairment compared to girls in the first years of their life [19], and also higher rates of behavior problems than girls in the later years following adoption [42].

There was no association between depressive symptoms of the mother and the EA. This was contrary to our hypothesis and one explanation for this may be that the scores of maternal depressive symptoms were low and a bigger sample size would have been needed to evaluate these associations. In studies, adoptive families have seemed to be well adjusted in terms of satisfaction with adoption, familial functioning, and parent–child communication; moreover, adoptive parents have not differed from birthparents in terms of their depressive symptoms after adoption [68, 69]. Still, it has been suggested that adoptive parents present symptoms of depression that can be difficult to detect for various reasons due to the exceptional setting of adoption [70]. In the future, more research is needed on the risk factors of the adoptive parent in their early interaction.

There are limitations to be considered in this study. First, the EAS are an observational measure based on a low-stress interaction for a relatively short period of time. Both the parent and especially older children can be conscious of the recording, which can make it more difficult to capture the whole nature of their interactions. Also, it can be argued that the laboratory setting can even contribute to this and a home observation could make the situation more natural and comfortable for the parent–child dyad. This could be true, especially in the unique situation of having a newly adopted child in the family. Nevertheless, EAS have shown adequate psychometric properties in 8–20 min of observation, regardless of the observational context [46, 47]. In their study, Ziv et al. examined the validity of the EAS against attachment security using a 10-min free play for EAS analysis and the Strange Situation Procedure for attachment measurement [26]. They found good construct validity between the two methods. In another study, Altenhofen et al. [25] found good convergent validity between maternal sensitivity, child responsiveness, child involvement and child attachment using the mother-reported Attachment Q-Set. Second, the aim of the study was to assess early interaction in the very beginning of the adoption and considering the first months after adoption a sensitive time, the assessments were planned to take place three months after the arrival of the child in the family. For various reasons, the time period between arrival of some children in their families and their interaction recordings were longer and the mean time from arriving in the family to the assessment was 6.3 months. Third, information about the background of the children was limited. It would have been of interest to examine the effect of different types of rearing conditions on the early interaction, but as most of the children had their background in institutions, this was not possible with the sample. More specific information on the type of care they have received prior to adoption would be valuable, as there are differences in rearing conditions between institutions. However, reliable information on these background factors is limited. Fourth, our study included only mother–child dyads as there were too few father-child dyads available, and no information about the number of possible siblings was included. In the future, more study is needed on the interaction of the child with both adoptive parents and on the effect of the structure of the adoptive family on the interaction. Fifth, although the EA has been evaluated to be a psychological construct in order to measure universal features in a parent–child relationship, there may be cultural aspects that influence the EA [21, 71]. However, the cultural factors that often influence parenting, like beliefs and norms [72], are in Finland quite similar to those in the major countries that receive internationally adopted children. Moreover, differences in parenting within cultures may often be larger than between cultures [72], which emphasizes the effect of background factors like socioeconomic status of the family and parent gender and age on parenting rather than country-specific cultural factors. As for depressive symptoms, there is some variability between countries. In Finland, the prevalence of depression is quite close to the prevalence of depression in the major countries that receive internationally adopted children [73, 74]. Sixth, the relatively small size of our sample makes a limitation to the study, as smaller differences between the groups may have been detected with a larger sample size. As there was a small number of children adopted from South-America and Eastern Europe, we had to combine these groups for the regression analysis even if this is a very heterogenous group. Due to the these low numbers, the potential differences between continents of birth were not possible to reliably analyze. Finally, the cross-sectional design of our study can be a limitation. As the first months in the family can be estimated to be a period of rapid development, examining only one time point gives us very limited knowledge of the process. Also, as depressive symptoms of the parent can be hard to detect in this period soon after adoption, the evaluation could have been done before the placement of the child in the family. Thus, more study in a longitudinal setting is needed on the development of early interaction.

## Summary

Although most adoptive families are well adjusted, internationally adopted children are overrepresented in mental health services and their problems can be complex and challenging. In addition to contributing to the understanding of their early interaction, our results can help in planning future treatment and support for this high-risk population. Our study suggests that families with post-institutionalized internationally adopted children are generally able to create a well-functioning early relationship between the mother and the child, even with the child presenting with some challenging behavior. Considering these challenges in childrens’ behavior, adoptive families may benefit from support in parenting in the first months after adoption. Our results also suggest that families with children adopted at a younger age as well as families with a boy may have more difficulties with the interaction in the first six months and especially these groups could benefit from family interventions in the early stage after adoption.

## Data Availability

The data presented in this article are not readily available because this article is based on health data and the data cannot be shared publicly because of GDPR, local protection act. Access to this data is regulated by Finnish legislation and FinData, and the Health and Social Data Permit Authority. The disclosure of data to third parties without explicit permission from Findata is prohibited. Only those fulfilling data the requirements established by Finnish legislation and Findata for viewing confidential data are able to access the data. For further information and data access queries, please visit to https://www.findata.fi/en/about-us/data-protectionand-the-processing-of-personal-data/.
